# Robust acoustic representation of Japanese vowels under phonation variability using dynamic spectral analysis

**DOI:** 10.1371/journal.pone.0361125

**Published:** 2026-10-23

**Authors:** Yanlong Zhang, Tianxiang Cao, Yueheng Liu

**Affiliations:** School of Japanese Culture and Economics, Xi’an International Studies University, Xi’an, China; University of Missouri Columbia, UNITED STATES OF AMERICA

## Abstract

Phonation-induced acoustic variability, particularly in whispered speech, poses persistent challenges for stable vowel representation. Conventional static formant-based descriptors often fail to capture the dynamic spectral behavior of vowels under such conditions. This study evaluates the robustness of acoustic feature representations for Japanese vowels across normal and whispered phonation, comparing static formant measures with dynamic spectral features derived from Vowel Inherent Spectral Change (VISC). Dynamic features are used to characterize continuous formant trajectory patterns, while Support Vector Machine (SVM) and Random Forest (RF) classifiers serve as analytical probes to assess representation stability rather than as targets of algorithmic optimization. Results demonstrate that incorporating dynamic spectral information leads to more consistent vowel discrimination across phonation types compared to static-only representations, with similar performance trends observed across classification frameworks. These findings suggest that differences in classification outcomes are primarily driven by properties of the acoustic representations rather than by model choice. The study highlights the im-portance of representation-level design for robust speech signal analysis under phonation-induced variability and supports the use of low-dimensional dynamic features for vowel modeling in variable phonation conditions.

## 1. Introduction

Speech processing research fundamentally relies on acoustic representations to capture linguistically relevant information from complex speech signals [1–3]. Across a wide range of analytical contexts, the effectiveness of speech analysis depends not only on modeling techniques, but critically on how speech signals are encoded and structured in acoustic space [4,5]. While substantial progress has been made in modeling normally phonated speech, acoustic representations are often implicitly assumed to remain stable across different speech production conditions. This assumption, however, has not been sufficiently examined, despite long-standing evidence that changes in speech production mechanisms can substantially alter spectral structure and formant behavior [6,7]. Variability arising from non-normal phonation therefore raises fundamental questions about whether commonly used acoustic representations preserve their descriptive adequacy when phonation conditions deviate from normal speech [8]. Understanding the robustness of acoustic representations under production-induced variability remains a foundational issue for speech signal analysis.

Whispered and normal phonation differ substantially in both perceptual and acoustic properties. Whispered speech is produced through turbulent airflow without periodic vocal fold vibration, resulting in the absence of a fundamental frequency and a reduced harmonic structure [9]. These characteristics complicate acoustic analysis and pose persistent challenges for speech processing systems primarily designed for normal phonation. However, existing studies rarely address whether acoustic representations themselves remain stable under phonation-induced variability, leaving the mechanisms underlying representation robustness largely unexplored. Most prior work has evaluated whispered speech primarily in terms of classification performance, without explicitly examining whether commonly used acoustic representations remain stable when phonation conditions change [10–12]. As feature behavior may vary across phonation types and linguistic systems, commonly used acoustic descriptors may not preserve stable vowel representations under changing phonation conditions, and conclusions drawn solely from performance-based evaluations may therefore lack generalizability across languages or phonation modes. This issue is particularly salient for pitch-accent languages such as Japanese, which exhibit distinct prosodic and typological characteristics and have received comparatively limited attention in existing whispered speech research.

To address these issues, the present study examines how phonation-related variability affects vowel representation in acoustic space, with particular attention to languages with distinct prosodic structures. Focusing on Japanese as a representative pitch-accent language, vowel representations under normal and whispered phonation are analyzed using both static formant-based descriptors and dynamic spectral features. Statistical classifiers, including Support Vector Machines (SVM) and Random Forests (RF), are employed as analytical probes to evaluate the robustness of different acoustic representations across phonation conditions, rather than as targets of recognition optimization. Within this framework, the study investigates how phonation-induced variability influences acoustic feature behavior and the stability of vowel representations, with the aim of elucidating representation-level properties relevant to speech analysis under non-normal phonation.

Methodologically, this study treats statistical classifiers as analytical probes for examining acoustic representations, not as objects of algorithmic comparison. Differences in classifier performance are interpreted as reflections of feature behavior across phonation conditions, rather than only as indicators of recognition effectiveness per se. Within this framework, vowel representations under normal and whispered phonation are analyzed by comparing conventional static formant-based features with dynamic spectral features derived from Vowel Inherent Spectral Change (VISC). This design enables phonation-induced variability to be evaluated at the level of acoustic representation, without reliance on model-specific optimization.

Specifically, this study addresses the following research questions:

(1) How does phonation-induced variability reshape vowel representations in acoustic space when static formant measures are compared with dynamic spectral features?
(2) To what extent are observed patterns of vowel representation stability consistent across different classification frameworks when dynamic features are incorporated?
(3) How can classifier behavior be interpreted as evidence of representation robustness when evaluating low-dimensional acoustic features under small-sample conditions?

Based on prior findings on dynamic spectral behavior, phonation-induced variability is expected to differentially affect static and dynamic acoustic representations, with VISC-based features exhibiting greater stability across phonation types. Although absolute classification outcomes may vary across modeling frameworks, stable performance trends associated with dynamic features are hypothesized to reflect properties of the acoustic representations themselves rather than model-specific effects. Under this view, dynamic spectral features are expected to provide more consistent encoding of vowel structure in the presence of phonation-induced variability.

## 2. Literature review

The fundamental distinction between whispered and normal speech lies in the vibratory behavior of the vocal folds, which shifts from a closed, voiced state to a more open, voiceless configuration. In whispered speech, airflow passes through the vocal folds with reduced impedance, and frication replaces vocal fold vibration as the primary excitation source. This alteration in excitation mechanism substantially changes the acoustic characteristics of vowels, most notably through the attenuation or absence of fundamental frequency and a reduction in overall acoustic energy [12,13]. Additionally, because whispered speech requires greater articulatory precision to maintain intelligibility, it often exhibits a slower articulation rate and longer segmental duration than normally phonated speech [14]. The loss of salient acoustic cues such as fundamental frequency and harmonic structure poses significant challenges for speech analysis and modeling approaches developed primarily for normal phonation. Nevertheless, these phonation-induced differences establish an essential basis for examining how vowel representations vary—and potentially destabilize—across speech production conditions.

Despite substantial progress in speech recognition, machine learning-based classification of whispered speech remains relatively underdeveloped, particularly with respect to generalizability across phonation types and languages. Mature statistical learning algorithms such as RF [15] and SVM [16] have been widely adopted in speech processing due to their distinct modeling characteristics. Existing studies suggest that classifier behavior is sensitive to both acoustic conditions and the structure of feature representations. RF has often been applied in small-sample settings and in feature-rich or noisy environments [17], whereas SVM is commonly used for high-dimensional representations, although its behavior may be more affected by noise and data imbalance [18]. More specifically, RF has been reported to handle multi-class classification with relatively limited preprocessing, while SVM tends to perform stably when acoustic features exhibit clearer class separability, with outcomes depending on kernel choice and regularization strategies [19,20]. Hybrid approaches that integrate RF-based feature selection with SVM classification have also been explored as a means of reducing redundancy in high-dimensional feature spaces [21].

Importantly, under non-normal phonation conditions, differences in classifier behavior are more plausibly interpreted as reflecting sensitivity to phonation-induced acoustic variability in feature representations, rather than intrinsic superiority of a given algorithm. The absence of key acoustic cues such as fundamental frequency in whispered speech introduces additional variability that challenges the stability of acoustic representations and, in turn, influences how classifiers respond to those representations. In this context, observed variation in algorithmic outcomes cannot be straightforwardly attributed to model effectiveness without considering the representational consequences of phonation change. Moreover, most existing whispered speech studies have focused on non-tonal languages (e.g., English) or tonal languages (e.g., Mandarin Chinese), whereas pitch-accent languages such as Japanese, which rely on distinct prosodic mechanisms involving funda-mental frequency, remain comparatively underexplored. This typological gap further motivates systematic evaluation of acoustic representations and their robustness across phonation types within pitch-accent language contexts.

Existing research on whispered speech remains limited in the range of acoustic representations considered, a constraint that may affect the robustness of vowel modeling under variable phonation conditions. In studies of whispered Japanese speech, analysis has predominantly relied on static formant frequencies, particularly Formant 1 (F1) and Formant 2 (F2), measured at fixed temporal points within the vowel [22]. Empirical findings consistently show that whispered Japanese vowels exhibit elevated formant frequencies relative to normally phonated vowels, a pattern likewise reported for Chinese [23] and English [24]. While such static measurements effectively characterize broad spectral shifts associated with whispering, they provide only a partial account of vowel acoustics and do not fully reflect within-vowel spectral behavior.

Evidence from perceptual and acoustic research demonstrates that temporal variation within vowels plays a central role in speech perception. Experimental manipulation of English vowels shows that removing the steady-state portion results in only a minimal reduction in intelligibility, from 93.1% to 92.4%, whereas eliminating dynamic onset and offset portions leads to a substantially larger decrease, to approximately 86.8% [25]. This asymmetry has been consistently replicated in subsequent studies [26]. In addition, vowels produced by non-native speakers often exhibit increased temporal variability, including greater dispersion in formant trajectories and prosodic cues, which can further influence intelligibility, particularly under whispered conditions [27]. Taken together, these findings indicate that static midpoint representations do not sufficiently characterize vowel behavior when phonation-related acoustic variability is present, underscoring the necessity of incorporating dynamic acoustic representations in the analysis of whispered speech.

VISC is an acoustic representation designed to capture dynamic spectral variation within vowels and has been widely examined in studies of vowel perception and classification. In contrast to static features that describe formant characteristics at a single temporal point, typically the vowel midpoint, VISC characterizes spectral dynamics by analyzing formant trajectories at both the onset and offset of a vowel [25]. Dynamic spectral change is commonly characterized using two measures: vector length (VL), which captures the extent of formant displacement, and spectral angle (α), which reflects the overall direction of formant movement during the vowel [27,28]. Early studies demonstrated a strong correspondence between listeners’ perceptual identification of English vowels and confusion patterns derived from VISC-based formant measurements [29]. This relation-ship was subsequently supported by later investigations [30]. Even when substantial variation in surrounding consonantal context induces marked shifts in dynamic spectral patterns, vowel perception has been shown to remain predictable based on these dynamic features. These results point to the importance of formant information near vowel boundaries, which may be underrepresented when vowels are described solely by static mid-point values.

A recent study examined the applicability of VISC to whispered vowel classification [31]. The authors conducted a preliminary investigation that refined the measurement procedure of VISC and evaluated its effectiveness relative to conventional static formant features across different phonation types and speaker genders. Using a SVM as the sole classification framework, the study reported that whispered vowels exhibited reduced vector length compared with normally phonated vowels, while spectral angle showed relatively limited variation across phonation types. In addition, the authors observed that dynamic features appeared less sensitive to speaker gender. When VISC features were combined with static formant measures, classification accuracy improved by approximately five percent compared with models relying on static features alone.

Despite these initial findings, the extent to which the reported advantages of VISC can be interpreted as properties of the acoustic representation itself remains constrained by several methodological factors in the study [31]. Specifically, the use of a single classification algorithm restricted evaluation of the observed advantages of dynamic features across different modeling frameworks. Furthermore, the speech material consisted of repeated productions of a fixed text, yielding a relatively small and homogeneous dataset. Moreover, as VISC is derived solely from the trajectories of F1 and F2, it provides a comparatively low-dimensional acoustic description relative to feature sets such as Mel Frequency Cepstral Coefficients (MFCCs). Given these constraints, it remains unclear whether the observed benefits of VISC reflect intrinsic properties of the acoustic representation itself or are partially dependent on the specific classifier employed. These considerations motivate further examination of the stability of VISC-based vowel representations across different classification frameworks and phonation conditions, particularly under phonation-induced acoustic variability.

## 3. Materials and methods

### 3.1.Speech samples

The data utilized in this study were sourced from the Japanese Versatile Speech (JVS) Corpus. This corpus is a freely accessible, publicly available dataset. Further details of which can be found on the introductory webpage mentioned in [32]. The corpus contains approximately 30 hours of professionally recorded speech from 100 native Japanese speakers, all of whom are trained voice actors. The corpus contains recordings in three phonation types: normal, whispered, and falsetto. Recordings were captured in a professional studio at a 48 kHz sampling rate, then downsampled to 24 kHz using the Speech Signal Processing Toolkit (SPTK), and saved in 16-bit RIFF WAV format. For this study, 60 stimuli were randomly selected from the five shared sentences, comprising recordings from 30 speakers (15 male and 15 female) across two phonation types (whispered and normal), ensuring coverage of all five Japanese vowel categories, as shown in Table 1. This sampling strategy was designed to minimize the potential confounding effects of inter-speaker variability and to ensure consistent, high-quality manual vowel segmentation. The final dataset comprises 2410 manually verified vowel tokens across both phonation types, covering all five Japanese vowels in a range of consonantal environments. This sample provides a suitable basis for the supervised classification comparisons at the core of the study.

**Table 1:** Japanese speech stimuli and corresponding IPA transcriptions. Five sentences sourced from the JVS corpus, covering diverse phonetic contexts and including all five Japanese vowels.

Sample No. and English translation	IPA of Japanese texts
VOICEACTRESS100_001: *In many cases, as seen at Tō-ji Temple, it is also placed at the center among the principal Myōō deities known as the Five Great Wisdom Kings.*	/mata toRdʑi no joRni ɡodai m^j^oRoR to jobaɾeɾɯ ɕɯjoRna m^j^oRoR no tɕɯRoRni haisaɾeɾɯ koto mo oRi/.
VOICEACTRESS100_002: *The New England style is a white cream soup based on milk, also known as Boston clam chowder.*	/n^j^ɯRiNɡɯɾando fuR wa ɡ^j^ɯRn^j^ɯR o beRsɯ to ɕita ɕiɾoi kɯɾiRmɯ sɯRpɯ de ari bosɯtoN kɯɾamɯ tɕaɯdaR to mo jobaɾeɾɯ/.
VOICEACTRESS100_003: *A category for individuals associated with computer game manufacturers or industry organizations.*	/koNp^j^ɯRta ɡeRmɯ no meRkaR ja ɡ^j^oRkai daNtai nado ni kaNɾeNsɯɾɯ dʑiNbɯtsɯ no kateɡoɾi/.
VOICEACTRESS100_004: *As this is a station where the service manager system has been introduced, it is remotely managed from Ōimachi Station.*	/saRbisɯ maneRdʑaR doRn^j^ɯReki no tame oRimatɕi eki kara eNkaku kaNɾi ɕite iɾɯ/.
VOICEACTRESS100_005: *By the time of the Silver Surfer attack incident, Richards had already become internationally recognized as both a superhero and a celebrity, along with the team name.*	/ɕirɯbaRsaRfaR ɕuRgekidʑikeN madeni ritɕaRdzɯwa tɕiRmɯmeRto tomoni kokɯsaitekini sɯRpaRçiRɾoR ojobi jɯRmeRdʑiNto ɕite niNtɕi saɾete iɾɯ/.

As the ethics statement, this study conducted acoustic analysis based on a publicly available, de-identified speech corpus. No new human participants were recruited, and no direct interaction with human subjects was involved in this research. The original data collection of the corpus was performed in accordance with relevant ethical guidelines, and informed consent was obtained from all participants at the time of data acquisition. According to the ethical review regulations of Xi’an International Studies University, secondary research using only publicly accessible, fully anonymized data with no risk to participant privacy is exempt from formal institutional ethical review. Therefore, formal approval from the Institutional Review Board was not required for this study. No additional informed consent was needed for the present secondary analysis.

### 3.2. Formant extraction

The analysis of both static and dynamic vowel features required measuring the F1 and F2 formant frequencies. Vowel boundaries were initially identified following the method described previously [12]. Automated extraction of F1 and F2 was performed using the acoustic analysis software Praat (version 6.3.09; https://www.fon.hum.uva.nl/praat/) and its Python API, Parselmouth (version 0.4.3; https://github.com/YannickJadoul/Parselmouth). Due to the lower signal-to-noise ratio of whispered speech, Praat’s default settings (which are optimized for normal speech) often produce noisy and unreliable formant tracks (Fig 1(a) and (b)). As a result, adjustments to analysis parameters, particularly linear predictive coding (LPC) coefficients, were necessary. To account for the distinct formant characteristics of whispered speech and reduce inter-speaker variability, formant-specific parameter settings were applied for each subject. By default, Praat sets the maximum number of formants to five. However, within the 0–5500 Hz frequency range, whispered vowels, which are characterized by elevated formant frequencies, typically exhibit only three detectable formants. Using the default setting may lead to the false detection of noise as additional formants. On the other hand, reducing the setting to two and limiting the “Formant ceiling (Hz)” to the F2 range risks excluding valid F2 values near the formant boundary, since Praat automatically filters out any formants above “Formant ceiling (Hz) - 50 Hz.” In this study, default values were retained for the “Time step (s),” “Window length (s),” and “Pre-emphasis from (Hz)” parameters. Adjustments were applied only to the “Maximum number of formants,” which was set to n + 1 (where n denotes the order of the target formant), and to the “Formant ceiling (Hz),” which was set to f + 100. This configuration ensured accurate formant identification in whispered speech without introducing spurious formants, yielding a notable improvement in extraction quality (Fig 1(c) and (d)).

**Fig 1:**
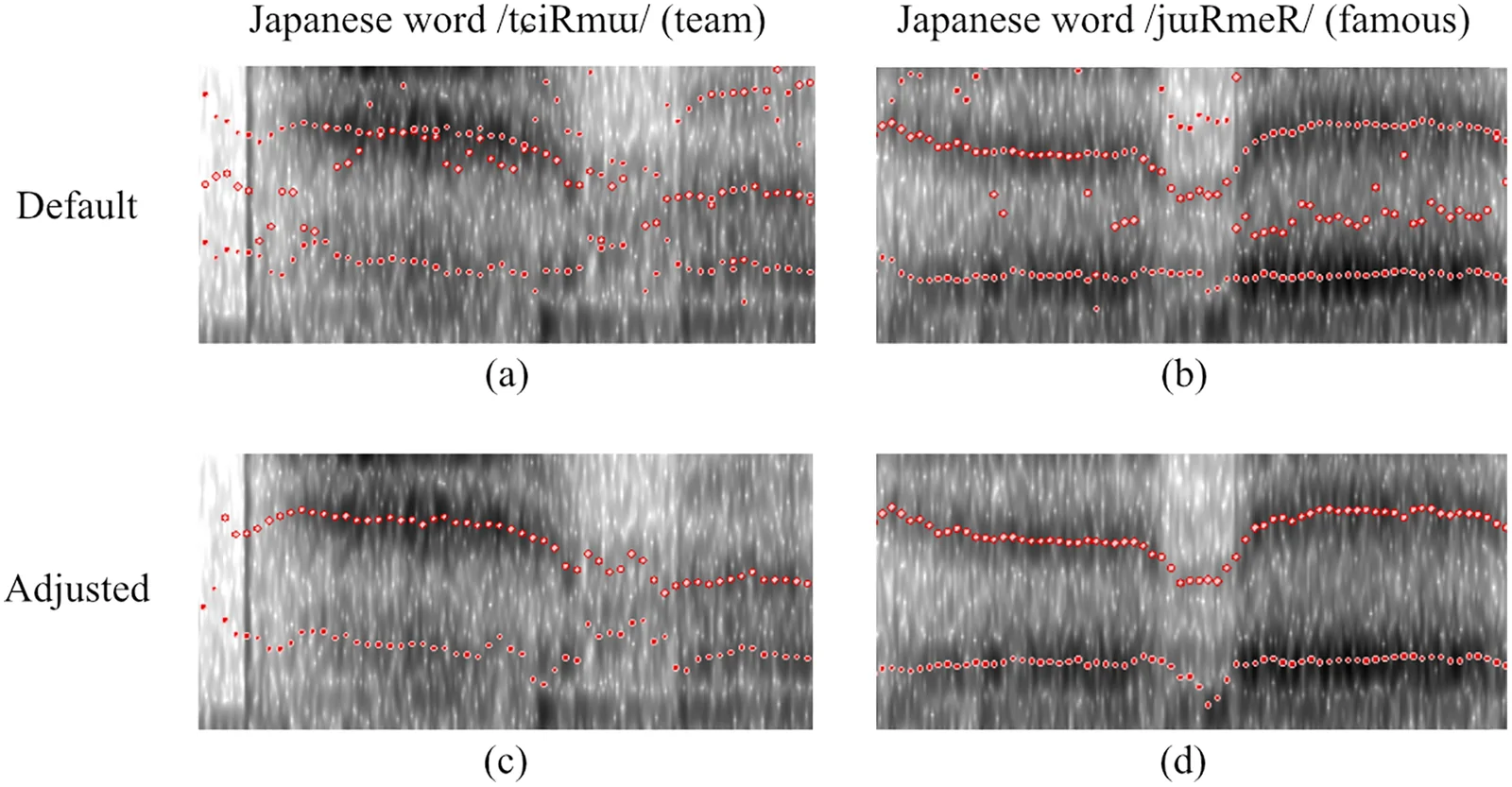
Spectrograms of whispered vowels using default (a) and (b) vs. optimized (c) and (d) Praat parameters. Adjusted settings (formant ceiling = f + 100 Hz; max formants = n + 1) eliminated spurious formants and improved trajectory clarity.

All vowel boundaries were manually annotated in Praat using standardized acoustic criteria. Vowel onset was defined by the concurrent emergence of clearly visible first and second formants and a rapid increase in vocalic amplitude, whereas vowel offset was identified at the point where formant structure weakened and transitioned to the following consonant. To assess segmentation reliability, kernel density estimation of vowel duration was computed for both phonation types (Fig 2). The distribution shows a primary peak at approximately 60 ms, corresponding to phonemic short vowels, and a secondary peak near 130 ms, reflecting phonemic long vowels. This bimodal duration pattern is consistent with the established temporal properties of Japanese vowels, and supports the validity of the segmentation.

**Fig 2:**
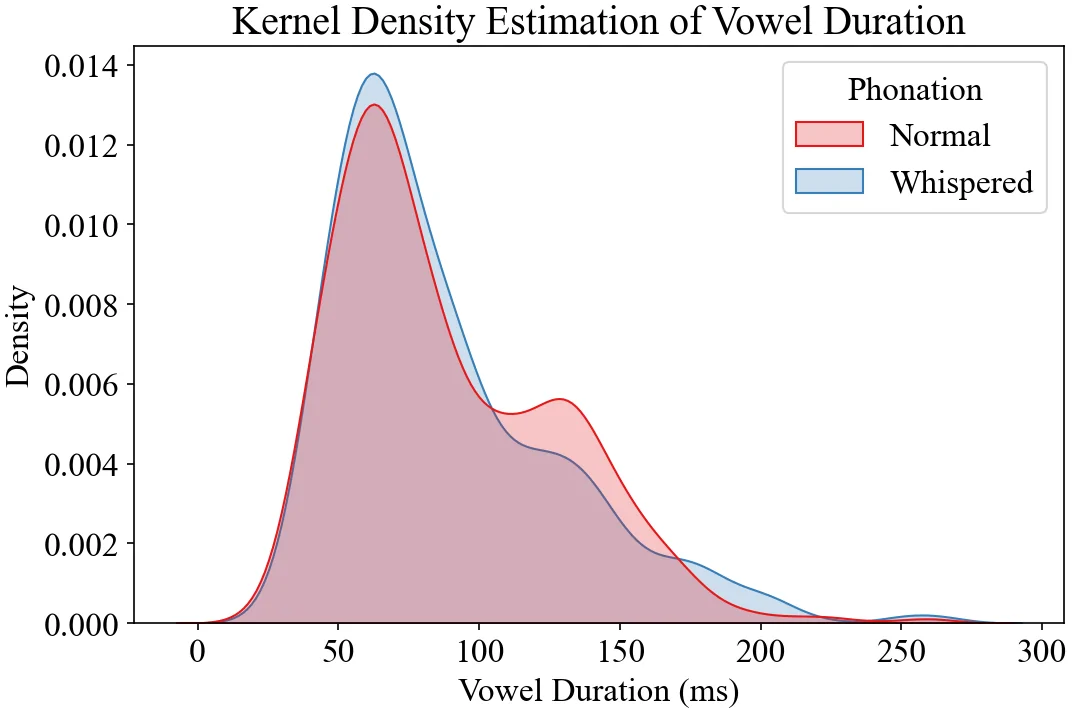
Kernel density estimation of vowel duration in normal and whispered speech. The primary peak around 60 ms corresponds to phonemic short vowels, and the secondary peak near 130 ms reflects phonemic long vowels in Japanese.

Given the inherent variability in formant frequencies, particularly in whispered speech, formant values were averaged over a segment centered on each vowel’s temporal midpoint. Our empirical testing determined that averaging across a 15ms window (7.5ms before and after the midpoint) provided optimal stability for both whispered and nor-mal speech. Additionally, 6.15% of vowel samples were excluded due to issues such as durations shorter than 20ms, unclear formant contours that impeded reliable extraction, or missing values resulting from formant ceiling configurations. These exclusions were necessary to preserve the validity of subsequent analyses.

No additional speaker-specific formant normalization was applied in this study. This is distinct from the per-subject optimization of formant extraction parameters, which was performed solely to ensure measurement reliability and is detailed earlier in this section. The core analysis compares the classification performance of static and dynamic spectral features within the same speaker cohort. Although inter-speaker variation in vocal tract length remains present, it is expected to influence all feature sets similarly; therefore, it is unlikely to confound the relative performance comparisons that constitute the study’s main conclusions. The potential influence of systematic sex-related differences in vocal tract scale was reduced through a sex-balanced sample comprising 15 male and 15 female speakers. F0-dependent vowel normalization methods were not applicable because whispered speech is produced without phonation and lacks a measurable fundamental frequency.

### 3.3. VISC measurement

For each vowel in both normal and whispered speech, VISC is characterized using Vector length (VL) and spectral angle (α). The calculation of them follows methodologies established and revised in prior research [29,30], as shown in Equations (1–4). VL, representing the magnitude of formant movement over the vowel’s duration, is defined as the Euclidean distance between the F1 and F2 frequencies measured at the 20% and 80% time points in the F1 × F2 plane. In contrast, α denotes the angular direction of this formant change, calculated using the atan2 function between those same time points. In these equations, ${F\text{1}}_{\text{8}\text{0}}$, ${F\text{1}}_{\text{2}\text{0}}$, ${F\text{2}}_{\text{8}\text{0}}$, and ${F\text{2}}_{\text{2}\text{0}}$ refer to the F1 and F2 frequencies at the 80% and 20% positions. By quantifying both the magnitude (VL) and direction (α) of spectral movement, these two metrics together offer a detailed representation of dynamic vowel articulation, capturing subtle but significant shifts in formant trajectories.


$$\begin{array}{cc} & VL=\sqrt{{\Delta F\text{1}}^{\text{2}}+{\Delta F\text{2}}^{\text{2}}}, \end{array}$$ (1)

$$\begin{array}{cc} & \Delta F\text{1}={F\text{1}}_{\text{8}\text{0}}-{F\text{1}}_{\text{2}\text{0}}, \end{array}$$ (2)

$$\begin{array}{cc} & \Delta F\text{2}={F\text{2}}_{\text{8}\text{0}}-{F\text{2}}_{\text{2}\text{0}}, \end{array}$$ (3)

$$\begin{array}{c} \begin{array}{c} \alpha =\text{atan2}\left(\Delta F\text{2},\Delta F\text{1}\right)=\;\left\{ \begin{array}{cccc} & {\text{tan}}^{-\text{1}}\left(\frac{\Delta F\text{2}}{\Delta F\text{1}}\right), & & \text{if }\Delta F\text{1}>\text{0}\\ & {\text{tan}}^{-\text{1}}\left(\frac{\Delta F\text{2}}{\Delta F\text{1}}\right)+{\text{180}}^{\circ }, & & \text{if }\Delta F\text{1}<\text{0}\text{ and }\Delta F\text{2}\geq \text{0}\\ & {\text{tan}}^{-\text{1}}\left(\frac{\Delta F\text{2}}{\Delta F\text{1}}\right)-{\text{180}}^{\circ }, & & \text{if }\Delta F\text{1}<\text{0}\text{ and }\Delta F\text{2}<\text{0}\\ & {\text{90}}^{\circ }, & & \text{if }\Delta F\text{1}=\text{0}\text{ and }\Delta F\text{2}>\text{0}\\ & -{\text{90}}^{\circ }, & & \text{if }\Delta F\text{1}=\text{0}\text{ and }\Delta F\text{2}<\text{0} \end{array}.\;\right. \end{array} \end{array}$$ (4)

Fig 3 provides a visual representation of the aforementioned calculations, illustrating the temporal dynamics of F1 and F2 frequencies. The left panel (a) depicts the F1 and F2 values at the 20% and 80% time points of the vowel’s duration, represented by circular and triangular markers, respectively. The right panel (b) presents the results of the VL and α calculations within the F1 × F2 plane. Each point on the plane corresponds to the F1 and F2 frequencies at a specific time point, while arrows indicate the vectors connecting the formant values at the 20% (F1_20_, F2_20_) and 80% (F1_80_, F2_80_) positions. The angle α quantifies the direction of formant movement, defined as the angle between this vector and the F1 axis.

**Fig 3:**
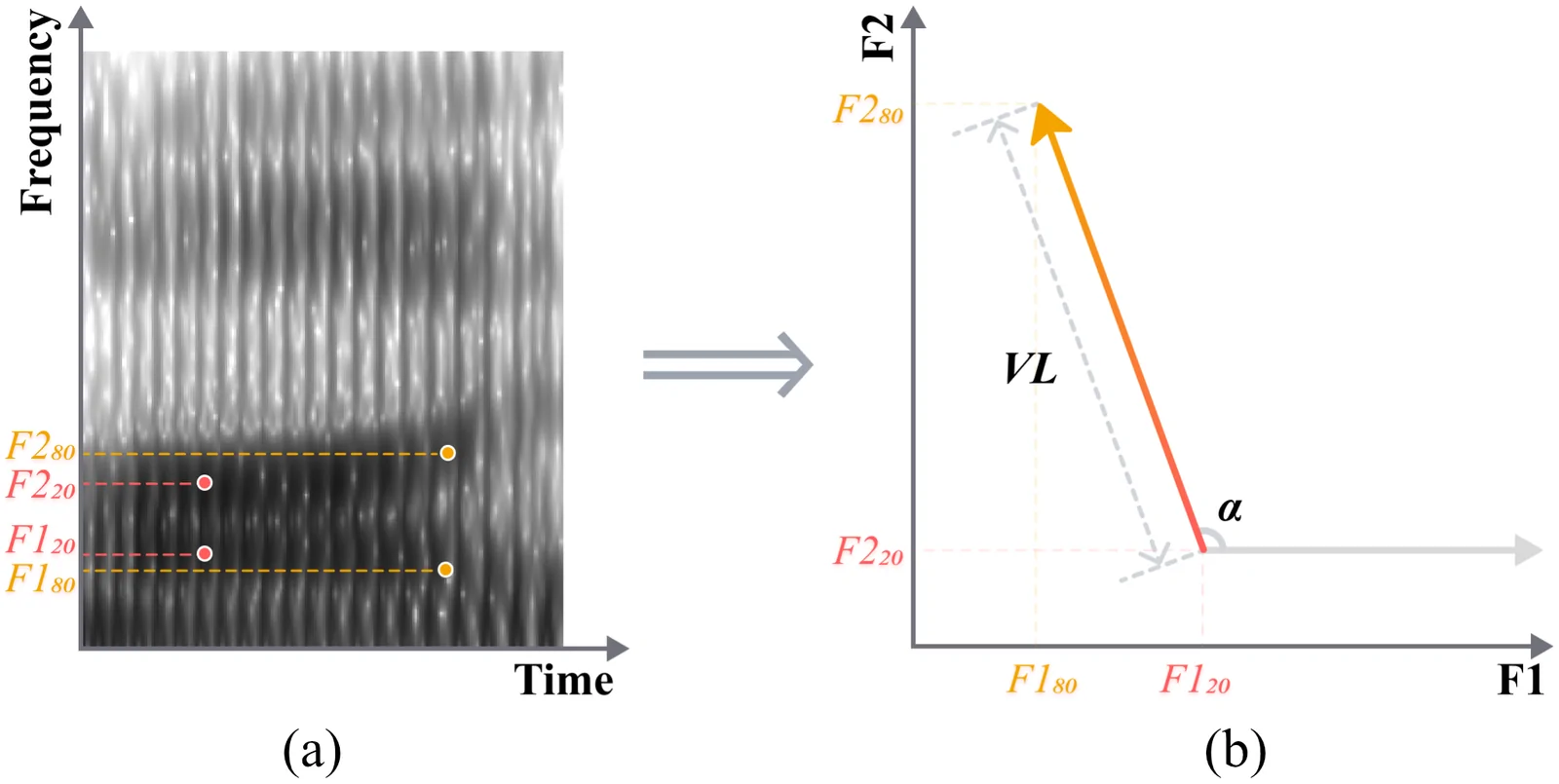
Visualization of VISC metrics. (a): F1/F2 values at 20% (circles) and 80% (triangles) of vowel duration. (b): VL (magnitude) and α (direction) in F1 × F2 space. Arrows denote formant trajectories between timepoints.

To facilitate a more intuitive understanding of the relationship between the two VISC parameters, we employed GeoGebra software to generate a dynamic visualization illustrating their interplay. Fig 4 depicts the variation of VL and α under contrasting formant patterns. Specifically, a marked difference in both VL and α is observable when changes in F1 and F2 exhibit similar trajectories (a) compared to when their directions diverge (b).

**Fig 4:**
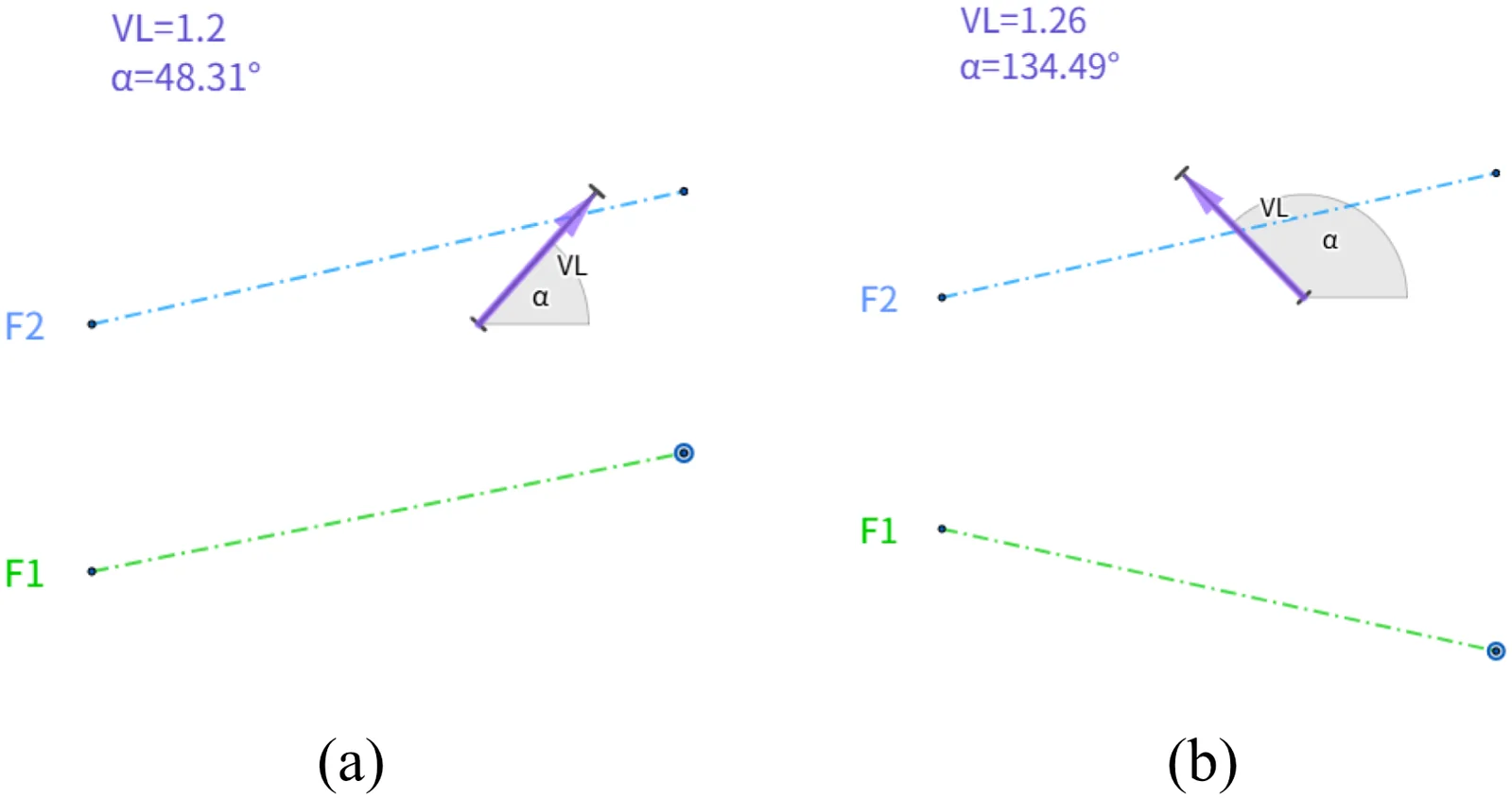
Dynamic GeoGebra visualization of VL and α under contrasting formant patterns. Convergent shifts (a) vs. divergent shifts (b). Interactive version: https://www.geogebra.org/calculator/dsptqk4e.

All acoustic measurements generated in this study have been deposited in the public repository “figshare”. The dataset includes static midpoint F1 and F2 values, VISC parameters (vector length and spectral angle) for all vowel tokens, as well as corresponding speaker information (participant ID and sex) and phonation condition labels. The dataset is available at https://figshare.com/articles/dataset/Formant_F1_F2_and_VISC_Features_of_Japanese_Whispered_and_Normal_Vowels/33307449. https://doi.org/10.6084/m9.figshare.33307449.

### 3.4.Statistical analysis

Initially, in addition to measuring the VISC dynamic features, descriptive statistics included the calculation of average F1 and F2 values for the five Japanese vowels /a/, /i/, /ɯ/, /e/, and /o/ in both normal and whispered speech, representing traditional static features. From these values, we also computed the formant displacement ratio (R) between the two phonation types, as shown in Equation (5), where $F_{whispered}$ and $F_{normal}$ represent the formant frequencies for whispered and normal speech, respectively.


$$\begin{array}{c} \begin{array}{c} R=\frac{F_{whispered}}{F_{normal}}\times \text{1}\text{0}\text{0}\%, \end{array} \end{array}$$ (5)

Subsequently, building on these measurements, vowel classification experiments were conducted to examine how dynamic features contribute to the stability of vowel representation across phonation types. The dataset was divided into two subsets based on phonation type: normal and whispered speech. For each subset, three distinct feature sets were evaluated:

- SF (Static Features): Only F1 and F2 were used to train RF and SVM classifier models independently;
- DF (Dynamic Features): Only VL and α were used to train RF and SVM classifier models independently;
- SF + DF (Combined Features): F1, F2, VL, and α were combined to train RF and SVM classifier models independently.

For both the RF and SVM classifier models, hyperparameters were initialized to their default settings (RF: n_estimators = 100, criterion = “gini”, max_features = “sqrt”; SVM: kernel = “rbf”, C = 1, gamma = “scale”). The dataset was partitioned into training (70%) and testing (30%) subsets via random sampling and subjected to iterative training. Model performance was assessed using a 5-fold cross-validation strategy, carefully designed to prevent data from the same speaker from appearing in both the training and testing sets within any fold. To ensure robustness and reproducibility, this process was repeated across 50 iterations, each using a unique random seed defined as 42 + i, where i corresponds to the iteration number (ranging from 0 to 49).

## 4. Results

The results are organized to examine vowel representations under normal and whispered phonation from complementary analytical perspectives. Section 4.1 presents descriptive acoustic measurements. This section focuses on both traditional static formant features and the dynamic VISC features introduced in this study to characterize the distribution of Japanese vowels in acoustic space across phonation types. Section 4.2 then reports SVM and RF classification outcomes. These models are employed as analytical tools to assess how the acoustic representations behave across different modeling frame-works. Fig 5 provides an overview of vowel distributions in the F1 × F2 space for whispered and normal speech. As depicted, the confidence ellipses for whispered vowels exhibit greater overlap and dispersion than those for normal speech, indicating reduced separability of vowel categories based on static formant structure and motivating further analysis of representational robustness.

**Fig 5:**
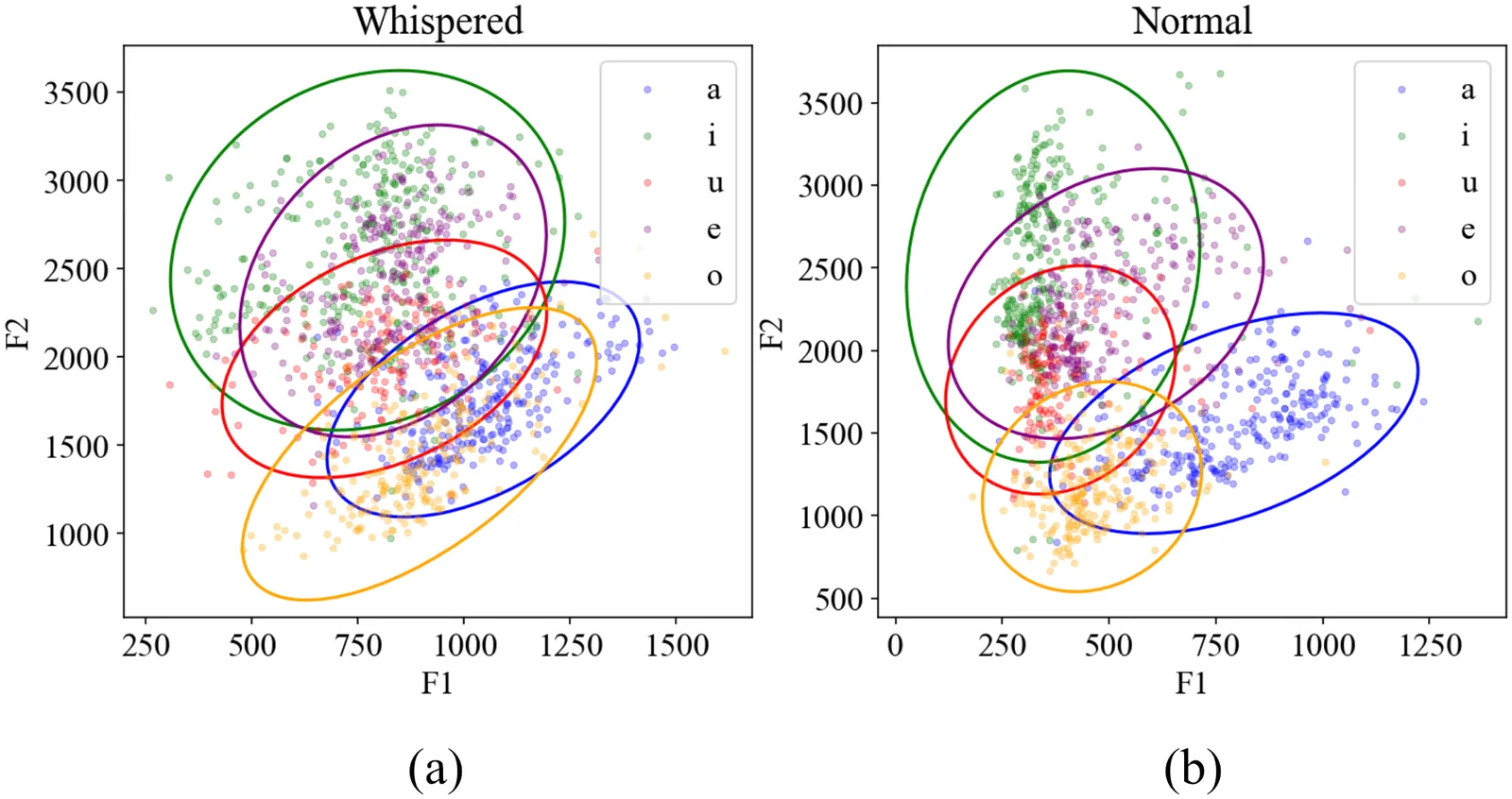
Acoustic distribution of Japanese vowels in F1 × F2 space under whispered (a) and normal (b) phonation. Confidence ellipses (95%) show greater overlap and dispersion in whispered speech, indicating reduced vowel separability.

### 4.1. Descriptive statistics

Table 2 summarizes the mean F1 and F2 frequencies of the five Japanese vowels in normal and whispered speech, and Table 3 reports the corresponding formant displacement ratios (RF1 and RF2) across phonation types. Across all vowels, whispered speech shows systematically higher formant frequencies than normal speech. While F1 shows substantial relative increases, with values ranging from 31% to 114% (*p* < 0.01), F2 exhibits considerably smaller increases, specifically between 4% and 25% (*p* < 0.05). This pattern is consistent with previous findings, which report an inverse relationship between absolute formant frequency and proportional displacement under whispered phonation [31]. Vowels with lower F1 values in normal speech, such as /i/ and /ɯ/ (364.27 Hz and 384.32 Hz), display the largest RF1 values (2.14 and 2.09). In contrast, vowels with higher F2 values, including /i/ and /e/ (2505.74 Hz and 2289.17 Hz), show comparatively small RF2 values (1.04 and 1.06). Collectively, these results demonstrate that phonation-induced spectral shifts are non-uniform across both formants and vowels. This finding underscores the limitations of using static midpoint measurements to characterize vowel representations under whispered conditions.

**Table 2:** Mean formant frequencies (F1, F2; Hz) for five Japanese vowels (/a/, /i/, /ɯ/, /e/, /o/) in whispered and normal speech. Whispered vowels exhibited elevated F1 (↑31–114%) and F2 (↑4–25%) compared to normal speech.

Formant	Phonation type	Japanese vowels				
		/a/	/i/	/ɯ/	/e/	/o/
F1	Whispered	1046.41	780.15	806.23	832.81	896.56
	Normal	796.53	364.27	384.32	489.26	457.80
F2	Whispered	1763.11	2613.86	1972.85	2433.61	1462.04
	Normal	1568.76	2505.74	1816.02	2289.17	1165.71

**Table 3:** Formant displacement ratios (R = $F_{whispered}$ / $F_{normal}$ × 100%) for F1 (RF1) and F2 (RF2). Lower-frequency formants (e.g., /i/ F1) showed larger proportional increases.

Formant displacement ratio	/a/	/i/	/ɯ/	/e/	/o/
RF1	1.31	2.14	2.09	1.70	1.96
RF2	1.12	1.04	1.09	1.06	1.25

Tables 4 and 5 summarize the descriptive statistics of the VISC measures, VL and α, for the five Japanese vowels under normal and whispered phonation. Across vowels, whispered speech is consistently associated with shorter VL values compared to normal speech, indicating a reduction in the magnitude of formant movement over the vowel duration. This reduction is not uniform across vowels, but varies in extent. The most substantial differences are observed for /a/ (34.16 Hz, *p* < 0.01), followed by /ɯ/ (27.92 Hz, *p* < 0.01), /i/ (23.13 Hz, *p* < 0.05), and /o/ (17.44 Hz, *p* < 0.05), whereas /e/ exhibits only a minimal difference (1.37 Hz, n.s). In addition to these differences in VL, distinct directional patterns are observed for α across vowel categories. For /a/, /i/, and /e/, α values predominantly fall within the 0° to 90° range, reflecting concurrent increases in F1 and F2 between the 20% and 80% time points. In contrast, α values for /ɯ/ and /o/ span the range from 0° to –90°, indicating an increase in F1 accompanied by a decrease in F2 over the same interval.

**Table 4:** VL (Hz) of VISC for Japanese vowels. Whispered vowels had shorter VLs—indicating reduced spectral dynamics—compared to normal speech, with /a/ showing the largest difference (34.16 Hz).

Phonation type	/a/	/i/	/ɯ/	/e/	/o/
Whispered	164.65	201.56	173.54	182.89	197.70
Normal	198.81	224.69	201.46	181.52	215.14

**Table 5:** α (degrees) of VISC for Japanese vowels. Minimal differences between phonation types suggest direction-based stability.

Phonation type	/a/	/i/	/ɯ/	/e/	/o/
Whispered	27.95	9.25	−61.35	17.58	−16.61
Normal	28.58	7.14	−43.15	32.88	−34.69

### 4.2.Classification-based analysis of acoustic feature representations

To examine how acoustic feature representations behave under a commonly used classification framework, analyses were first conducted using SVM. Table 6 reports the average classification accuracy across all iterations and folds, and Figs 5 and 6 present the corresponding confusion matrices for individual vowel categories. For both normal and whispered speech, incorporating dynamic features (DF) together with static formant features (SF) led to a significant increase in classification accuracy (normal speech: t(98) = 9.18, *p* < 0.001; whispered speech: t(98) = 9.53, *p* < 0.001). As shown in Fig 6, the inclusion of dynamic features improved classification performance for vowels such as /a/ and /o/, with accuracy reaching 88%, and also resulted in enhanced accuracy for /i/ and /ɯ/, while improvements for /e/ were comparatively limited. A similar pattern was observed for whispered speech in Fig 7, where dynamic features increased classification accuracy by more than 5% for /ɯ/ and /o/. When only dynamic features were used, classification accuracy declined substantially in both phonation conditions, as illustrated in Figs 5(b) and 6(b). These results indicate that dynamic features alone are insufficient for reliable vowel categorization, but provide complementary information that enhances vowel representations when combined with static formant features.

**Table 6:** Mean classification accuracy (%) of SVM using SF vs. SF + DF. SF + DF significantly improved accuracy in both normal (79.20% vs. 75.60%) and whispered speech (62.71% vs. 58.65%; *p* < 0.001).

Dataset	SF	SF + DF
Normal	75.60	79.20
Whispered	58.65	62.71

**Fig 6:**
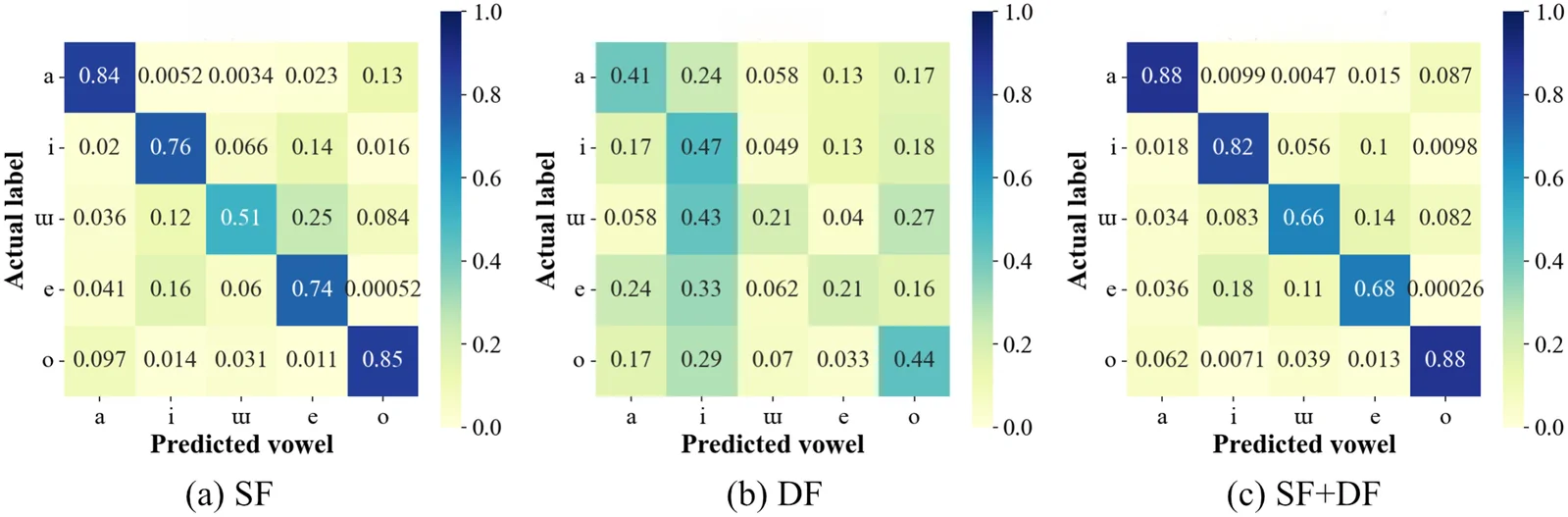
Confusion matrices for vowel classification in normal speech using SVM with: (a): SF (F1, F2); (b): DF (VL, α); (c): Combined features (SF + DF). Accuracy improved significantly with SF + DF (*p* < 0.001).

**Fig 7:**
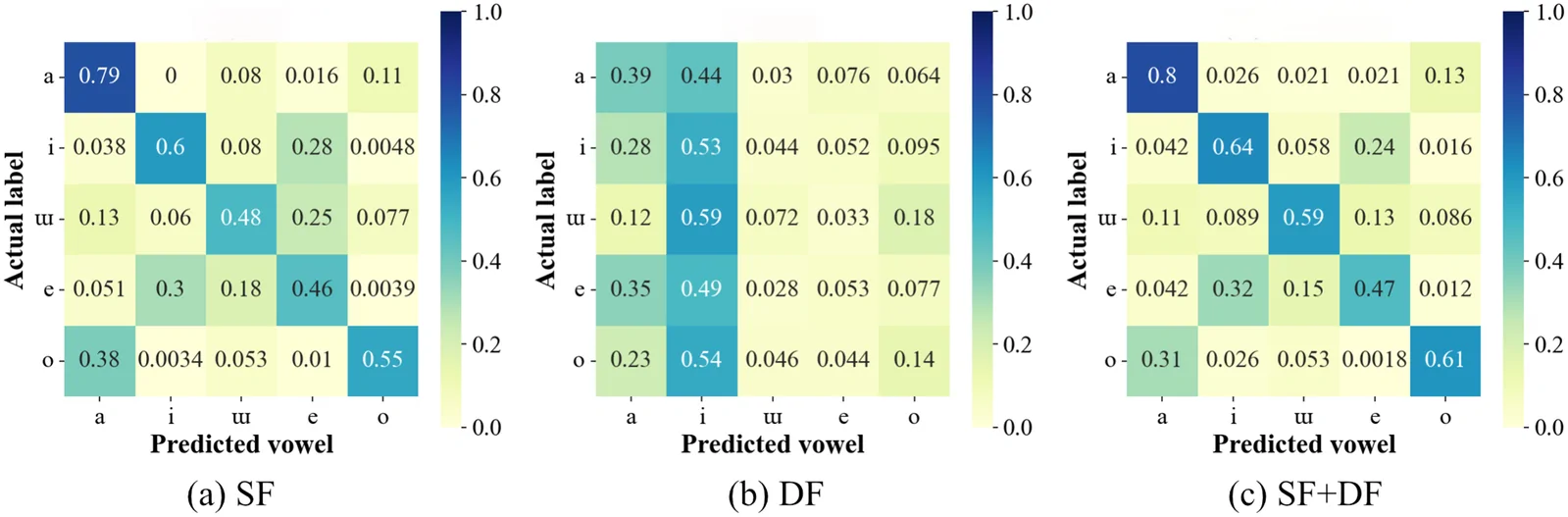
Confusion matrices for vowel classification in whispered speech using SVM with: (a): Static features (SF: F1, F2); (b): Dynamic features (DF: VL, α); (c): Combined features (SF + DF). SF + DF increased accuracy by >5% for /ɯ/ and /o/ (*p* < 0.001).

To examine acoustic feature representations under an alternative classification framework, analyses were conducted using RF models. Table 7 reports the average classification accuracy across all iterations and folds, and Figs 7 and 8 present the corresponding confusion matrices for individual vowel categories. Similar to the SVM-based analyses, the incorporation of dynamic features alongside static formant features yielded significant improvements in classification accuracy for both normal speech (t(98) = 13.30, p < 0.001) and whispered speech (t(98) = 12.64, p < 0.001). As depicted in Fig 8, the inclusion of dynamic features reduced misclassifications of /i/ and /e/ and increased the classification accuracy of /ɯ/ by approximately 15% compared with the static-feature condition. In whispered speech (Fig 9), dynamic features likewise reduced misclassifications involving /ɯ/, particularly with /e/, and improved classification accuracy for /a/ and /e/. Overall classification accuracy for RF was marginally lower than that observed for SVM. When only dynamic features were used, a decline in classification performance was also observed.

**Table 7:** Mean classification accuracy (%) of RF using SF vs. SF + DF. SF + DF enhanced accuracy in normal (78.09% vs. 72.40%) and whispered speech (61.34% vs. 56.19%; *p* < 0.001), though RF underperformed relative to SVM.

Dataset	SF	SF + DF
Normal	72.40	78.09
Whispered	56.19	61.34

**Fig 8:**
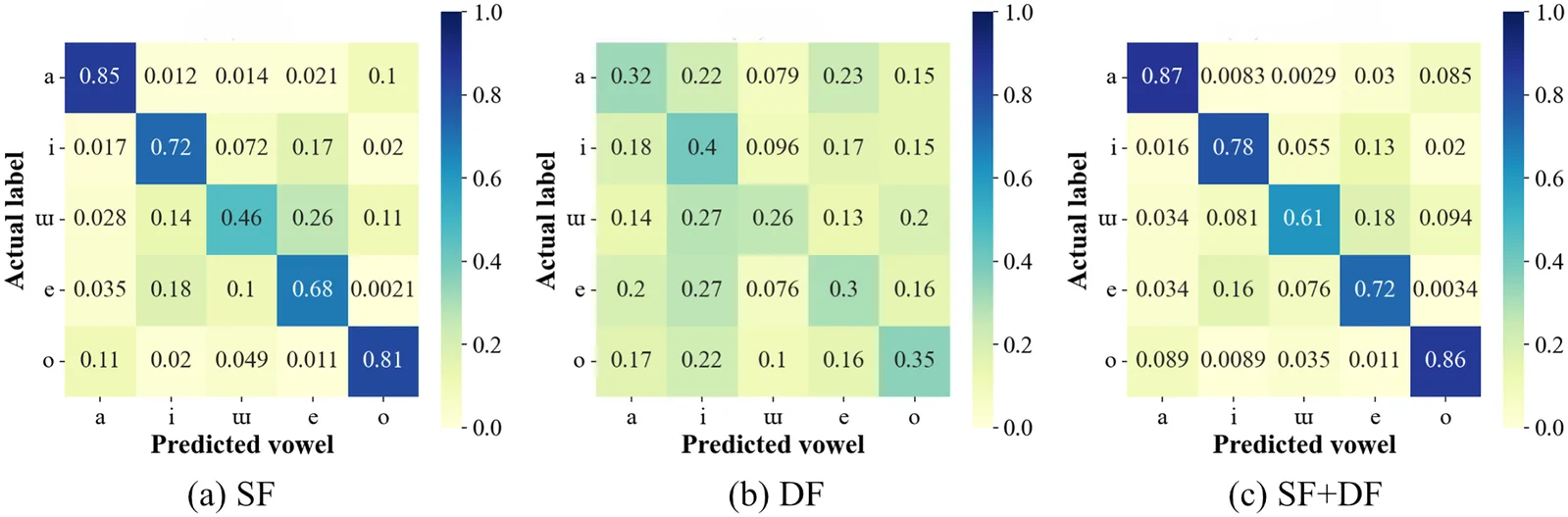
Confusion matrices for vowel classification in normal speech using RF with: (a): Static features (SF: F1, F2); (b): Dynamic features (DF: VL, α); (c): Combined features (SF + DF). SF + DF reduced misclassification of /i/ and /e/, and improved /ɯ/ accuracy by 15%.

**Fig 9:**
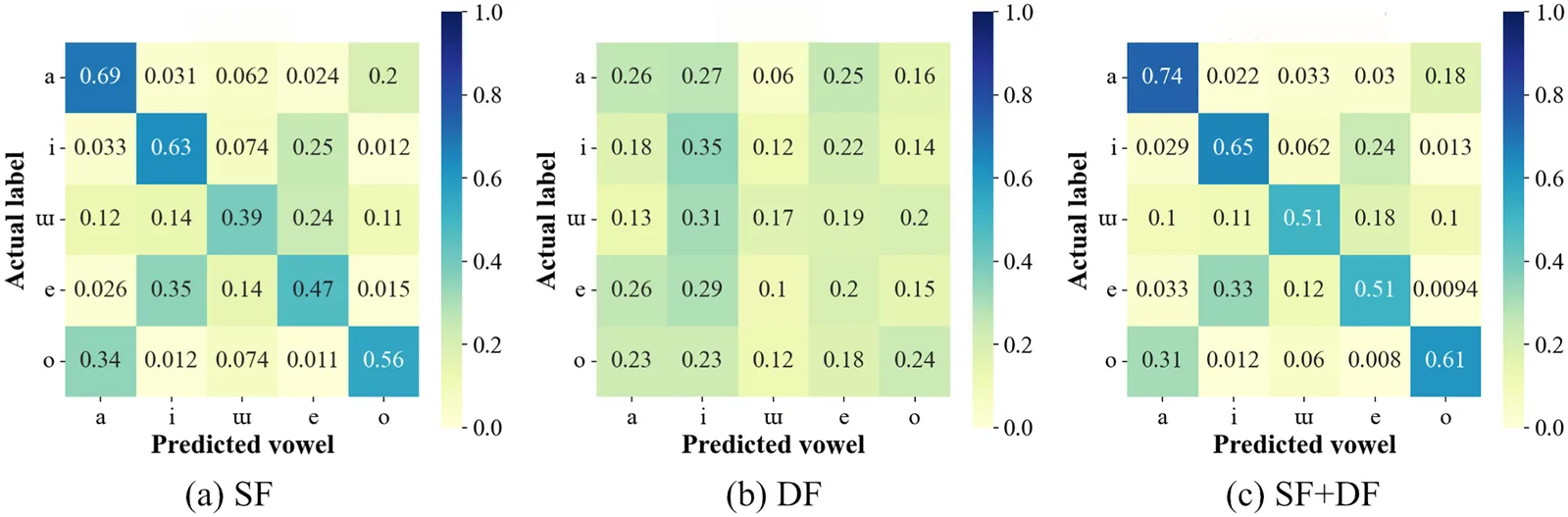
Confusion matrices for vowel classification in whispered speech using RF with: (a): Static features (SF: F1, F2); (b): Dynamic features (DF: VL, α); (c): Combined features (SF + DF). SF + DF mitigated /ɯ/–/e/ confusion and enhanced /a/ and /e/ accuracy.

To further elucidate how individual acoustic features contribute to vowel classification and to assess the interpretability of the RF models, a feature importance analysis was conducted using the SHAP (SHapley Additive exPlanations) framework with the TreeExplainer algorithm. For each vowel category and each phonation type, the mean absolute SHAP value was computed across all test samples and averaged over 50 independent iterations to eliminate sampling randomness. This metric quantifies the overall magnitude of each feature’s contribution to the model’s classification decisions, with larger values indicating greater influence on predictive outcomes.

Tables 8 and 9 summarizes the mean absolute SHAP values across 50 independent iterations. In normal speech, vowel identification is heavily dominated by static formant. In whispered speech, however, the contribution of F1 collapses substantially across all five vowels (with the mean SHAP dropping from 0.124 to 0.075) due to subglottal coupling through the abducted glottis and high-frequency aspiration noise. In sharp contrast, VISC demonstrate high relative robustness: while the importance of F1 drops sharply, the absolute SHAP values of α actually increase in /a/, /i/, and /ɯ/ (by 33%, 38%, and 29%, respectively), and the relative contribution of dynamic cues rises across all vowels. This confirms that the decision trees adaptively restructured their splits to prioritize formant trajectory dynamics as static spectral clarity degraded.

**Table 8:** Mean absolute SHAP values for each feature across five vowels in normal speech. Values represent the average magnitude of feature contribution to vowel classification, averaged across 50 iterations.

Feature	/a/	/e/	/i/	/o/	/ɯ/
f1	0.1997	0.1222	0.1427	0.0718	0.0820
f2	0.1241	0.1777	0.1892	0.2080	0.1031
VL	0.0129	0.0285	0.0269	0.0134	0.0178
α	0.0356	0.0461	0.0250	0.0339	0.0506

**Table 9:** Mean absolute SHAP values for each feature across five vowels in whispered speech. Values represent the average magnitude of feature contribution to vowel classification, averaged across 50 iterations.

Feature	/a/	/e/	/i/	/o/	/ɯ/
f1	0.1443	0.0630	0.0783	0.0328	0.0571
f2	0.1364	0.1431	0.2012	0.1868	0.0985
VL	0.0216	0.0254	0.0281	0.0205	0.0153
α	0.0474	0.0426	0.0346	0.0299	0.0654

Most notably, for the whispered vowel /ɯ/, the importance of α (0.0654) substantially surpasses that of F1 (0.0571). Empirical acoustic measurements clearly account for this rank reversal: First, whisper-induced upward shifts are highly asymmetric, exerting a disproportionate impact on lower frequencies. While F2 for /ɯ/ only slightly increases by 8.6% (1816.02 Hz to 1972.85 Hz), its F1 surges by 109.8% (from 384.32 Hz to 806.23 Hz), compressing the static F1 of /i, ɯ, e, o/ into an indistinguishable narrow band (780–896 Hz) and neutralizing F1 as a height indicator. Second, α surpasses F1 exclusively in /ɯ/ because other vowels retain a distinctly highest or lowest value in at least one formant dimension—/a/ has the highest F1 (1046 Hz), /i, e/ have the highest F2 (>2400 Hz), and /o/ has the lowest F2 (1462 Hz). In contrast, /ɯ/ has both its F1 and F2 situated in intermediate, overlapping ranges. With static formants failing to provide clear boundaries, the trajectory angle α, which remains robust against baseline frequency shifts, serves as an essential compensatory cue for distinguishing /ɯ/.

## 5. Discussion

From a speech engineering perspective, this study evaluates the robustness of acoustic feature representations for vowel modeling under phonation-induced variability using static and dynamic spectral descriptors. Across all conditions, dynamic VISC features improved vowel separability when combined with static formant measures, and similar performance patterns were observed for both SVM and RF models. These results indicate that classification differences are primarily driven by the properties of acoustic representations rather than by model choice, underscoring the importance of representation-level design for robust speech processing under phonation variability.

This experiment shows that integrating dynamic acoustic features with static formant measures improves vowel classification performance across different phonation types. The inclusion of VISC features resulted in accuracy gains exceeding five percentage points for both SVM and RF models and was accompanied by a reduction in confusions among several vowel categories. These improvements indicate that dynamic spectral information captures aspects of vowel structure not fully represented by static midpoint measurements alone. This finding contrasts with much of the prior research on vowel classification, which has primarily focused on improving recognition accuracy through the optimization of static acoustic features, such as midpoint formant frequencies and their derived representations [33–36]. While effective under normal phonation, such approaches offer limited insight into how vowel representations behave when phonation conditions change. The present findings instead suggest that dynamic spectral features contribute to more stable vowel representations across normal and whispered speech, proving especially valuable where phonation-induced variability disrupts static formant structure. Building on earlier work demonstrating the usefulness of VISC for whispered Japanese vowel classification using SVM [31], the present study extends this line of research by showing that the benefit of incorporating VISC is not confined to a single classification framework. Importantly, the results indicate that optimal classification performance is obtained only when dynamic features are integrated with static formant information.

Although both SVM and RF benefited from the inclusion of VISC dynamic features, RF consistently produced lower classification accuracy across feature sets and phonation conditions. This outcome does not indicate an inherent limitation of RF, but reflects how the two models interact with the acoustic feature space used in the present study. The feature sets were low-dimensional, consisting of F1 and F2 in the static condition and four parameters (F1, F2, VL, α) when dynamic features were included. In such settings, SVM can form stable decision boundaries through margin maximization and kernel-based mapping, which is known to be effective for non-linearly separable data [37,38]. RF, by contrast, relies on ensemble learning across decision trees and typically benefits from higher-dimensional feature spaces, where random feature selection helps reduce overfitting [15,39]. When feature dimensionality is limited, this advantage is less pronounced. After the inclusion of VISC features, the accuracy gap between SVM and RF decreased from 2.46% to 1.37%. This reduction suggests that dynamic acoustic information allowed RF to make more effective use of the available feature space and reduced model-dependent performance differences.

As a further methodological cross-check, we conducted supplementary classification experiments using 12-dimensional MFCCs as a baseline feature set, both alone and in combination with VISC-based dynamic measures. In line with expectations for general-purpose speech features, MFCCs yielded higher overall classification accuracy than formant-only features across both phonation conditions and both classification frameworks. However, the additional contribution of VISC measures was substantially smaller in the MFCC-based models than in the formant-based models. Specifically, for normal speech, SVM classification accuracy was 86.48% with MFCCs alone and increased to 87.22% after the addition of VISC features; for whispered speech, accuracy increased from 76.42% to 76.67%. Similar patterns were observed for RF classifiers: accuracy for normal speech increased from 82.19% to 82.75%, whereas accuracy for whispered speech increased from 74.58% to 75.29%. These modest improvements do not detract from the value of formant-based VISC analysis; they align with the study’s core findings. Crucially, the aim of the present study is not to achieve the highest possible classification accuracy using general-purpose acoustic features, but to clarify the acoustic-phonetic mechanisms underlying vowel dynamics under phonation-induced variability. From this perspective, the formant-based VISC framework remains essential, as it enables direct phonetic interpretation of the results, whereas MFCCs do not readily lend themselves to such mechanistic explanation. This supplementary analysis therefore serves as an independent corroboration of the dynamic nature of vowel representations and supports the robustness of our core conclusions across feature types.

While the integration of VISC with static formant features consistently improved classification accuracy, the relatively poor performance observed when using VISC alone requires further consideration. One contributing factor is that the RF and SVM models employed in this study are static classifiers, and they are not explicitly designed to model temporal dependencies. Although VISC reflects dynamic formant movement, it is operationalized as summary parameters instead of an explicit temporal sequence. In contrast, sequence-based architectures such as Long Short-Term Memory (LSTM) networks are specifically designed to capture temporal structure and be more effective for modeling time-dependent acoustic patterns [40]. In addition, the current VISC extraction procedure relies on endpoint-based formant differences or linear slopes, which may oversimplify vocal tract dynamics and fail to capture non-linear or fine-grained temporal variation. Previous work suggests that richer time–frequency representations, including approaches based on dynamic time warping or higher-order derivative features, can better reflect articulatory dynamics [41].

From a cross-linguistic perspective, the interpretation of VISC patterns must be situated within the phonological properties of the target language. Most foundational VISC research has been conducted on Indo-European languages such as English, which exhibit prominent diphthongization and substantial inherent spectral change. Japanese, by contrast, is a predominantly monophthongal language with no phonemic diphthongs. Crucially, however, the absence of diphthongization does not imply the absence of VISC. As established in seminal work on VISC [29], vowel inherent spectral change arises not only from phonemic diphthongal targets but also from the continuous dynamics of vocal tract articulation and coarticulatory influences from neighboring consonants, both of which are universal properties of speech production. This view is further reinforced by comprehensive reviews demonstrating that dynamic spectral cues contribute systematically to vowel identification even in languages without phonemic diphthong contrasts [25]. Accordingly, Japanese vowels would be expected to exhibit measurable but comparatively modest VISC magnitudes relative to languages with stronger diphthongal tendencies. This expectation is supported by our descriptive data: across all five Japanese vowels, vector lengths in normal speech fall within a narrow range of approximately 180–225 Hz, indicating that spectral dynamics are present but relatively compact in this vowel system.

It should also be noted that the 20–80% temporal window employed for VISC measurement, although standard in the literature, cannot fully eliminate coarticulatory effects from adjacent consonants. As demonstrated by [42], the extent of consonantal penetration into vowel intervals varies systematically across languages, such that the residual contextual influence captured within the 20–80% window is language-specific. Because the current stimuli were extracted from natural continuous utterances, the measured VISC values inevitably incorporate some coarticulatory information from neighboring segments. This property may contribute to the practical utility of VISC in real-world speech processing; however, it also means that the observed dynamics do not exclusively reflect vowel-internal articulatory targets. We acknowledge this issue as an inherent limitation of the present study design.

The typological characteristics of Japanese also help contextualize the finding that VISC features alone yield relatively low classification accuracy. In a compact five-vowel system with limited inherent spectral dynamics, where core categorical contrasts are defined primarily by static vowel height and backness dimensions [43], static formant frequencies already capture the primary phonological distinctions, whereas dynamic information functions mainly as a complementary cue rather than a primary discriminator [25]. This pattern is consistent with our classification results: dynamic features alone yielded substantially lower accuracy than static formant measures, yet they produced consistent improvements of 3.5–5.5% when combined with static features across both phonation types and both classifier frameworks. This pattern supports the view that the functional weight of dynamic vowel cues is partly dependent on the inventory size and phonological structure of a given language.

Dataset-related factors may also limit the effectiveness of VISC when used in isolation. The present analysis focused on the five Japanese vowels, which constitute a relatively compact vowel system with less pronounced formant transitions than those observed in languages such as English. Prior studies have shown that, for English vowels, steady-state spectral information alone can often provide sufficient contrast, thereby reducing the relative contribution of dynamic cues [44]. Moreover, although VISC is derived from a limited number of parameters, it reflects underlying formant trajectories and effectively increases representational complexity compared to static midpoint measures. Reliable modeling of such dynamic information generally requires larger training datasets to avoid overfitting and ensure robust generalization [45]. While the sample size in the present study is sufficient to support the reported findings, future work with expanded datasets will be necessary to exploit the potential of dynamic spectral features fully.

On a related note, the descriptive results of the VISC parameters in this study align with findings reported before [31]. Specifically, the VL for whispered Japanese vowels was found to be smaller than that observed in normal speech, suggesting a reduction in formant variability under whispered conditions. While the spectral angle α exhibited only minor differences between whispered and normal speech, this indicates that the direction of the VISC vector in the F1 × F2 space is relatively stable across phonation types. This robustness suggests that α holds considerable promise as a reliable acoustic parameter for complex speech engineering applications requiring resilience to variations in phonation. At the same time, the present results also suggest that, within the relatively compact Japanese vowel system examined here, the dynamic cues captured by VISC may be less salient or discriminative when used in isolation. Consequently, we hypothesize that, despite the limited efficacy of VISC in isolation, the dynamic and fine-grained acoustic features it captures are fundamentally important. Future research should therefore prioritize enhancing the alignment of temporal models, exploring dynamic non-linear feature extraction techniques, and addressing sample size considerations to ensure robust and accurate predictive performance within the study population.

In summary, this study investigates vowel representations under phonation-induced variability by integrating static formant measures with dynamic spectral features derived from VISC. The findings indicate that incorporating dynamic features alongside static information leads to more stable and discriminative vowel representations across both normal and whispered speech. These effects are consistent across classification frameworks, suggesting that performance differences mainly reflect the properties of the acoustic representations. Differences in classification performance across models can be attributed to how learning frameworks interact with low-dimensional acoustic representations. Overall, these findings underscore the critical role of representation-level modeling in achieving robust vowel classification across diverse phonation modes, including non-normal conditions such as whispered speech. By offering a balance of low dimensionality and high computational efficiency, the proposed features are well suited for real-time speech processing applications, particularly within the ecosystem of resource-constrained edge devices.
